# Asymmetric Connectivity Between Redox-Active Tyrosines and Reaction-Center Chlorophylls in Photosystem II

**DOI:** 10.3390/plants15172557

**Published:** 2026-08-22

**Authors:** Shalini Yadav, Dimitrios A. Pantazis

**Affiliations:** Max-Planck-Institut für Kohlenforschung, Kaiser-Wilhelm-Platz 1, 45470 Mülheim an der Ruhr, Germany

**Keywords:** photosystem II, redox asymmetry, tyrosine radicals, electron transfer, multiscale simulations

## Abstract

Photosystem II (PSII) contains several cofactors involved in light harvesting, charge separation, electron transfer, and catalysis. The initial charge separation in the reaction center of PSII creates the strongest known redox-cofactor oxidant in biology, a cationic radical distributed over a “special pair” of chlorophyll molecules (P680^•+^). Two redox-active tyrosines, Y_Z_ and Y_D_, located at opposite sides of the special pair, are the principal residues that reduce this cationic radical. Y_Z_, in turn, oxidizes the manganese cluster of the oxygen-evolving complex to drive water oxidation, whereas Y_D_ forms a stable radical facilitated by local water translocation. The details of this asymmetry and the role of nearby protein residues in mediating branch-specific electron/hole-transfer pathways remain incompletely understood. Here, we investigate pathways for electron transfer (ET) from Y_Z_ and Y_D_ to P680^•+^ and identify specific residues that are likely responsible for mediating ET. Graph-based analysis predicts aromatic residue-assisted pathways on both branches but also reveals a distinct tryptophan (D2-Trp191) that connects Y_D_ with P680^•+^, whereas the corresponding D1-side position is occupied by a non-aromatic D2-Ile192. This suggests a possible role of this tryptophan as an ET mediator, thereby differentiating the nature of electronic connectivity between Y_Z_/Y_D_ and the reaction center. Residue conservation analysis indicates retention of D2-Trp191 across various organisms. Molecular dynamics show that the predicted donor–mediator and mediator–acceptor contacts remain structurally persistent over the simulation, while QM/MM calculations show appreciable spin-density localization capacity, providing strong computational support for an ET mediator role of D2-Trp191. Together, these results suggest that ET between the redox-active tyrosines and the reaction-center chlorophylls occurs via distinct mechanisms—direct vs. mediated—with D2-Trp191 being a D2-specific mediator for the branch-selective electron/hole-transfer connectivity in PSII.

## 1. Introduction

Oxygenic photosynthesis is essential in sustaining life on earth [1,2]. The initial steps of oxygenic photosynthesis take place within photosystem II (PSII), where a sequence of light-triggered electron-transfer reactions among reaction-center pigments drive the four-electron oxidation of water to dioxygen and the two-electron reduction of plastoquinone to plastoquinol [3,4,5,6,7,8,9]. PSII exists as a dimeric membrane-bound complex. Among the transmembrane subunits, the highly conserved D1 and D2 proteins accommodate the chlorophylls and pheophytins of the reaction center, the manganese-containing catalytic site of water oxidation known as the oxygen evolving complex (OEC), and intermediate and terminal (mobile) electron-acceptor plastoquinones Q_A_ and Q_B_. The D1 (PsbA) and D2 (PsbD) proteins are surrounded by chlorophyll-rich proteins CP43 (PsbC) and CP47 (PsbB), which function as inner antenna proteins that transfer excitation energy to the reaction-center pigments. Several additional transmembrane subunits, including cytochrome b559 [10,11,12], complete the PSII complex, while, in addition to the transmembrane components, species-specific extrinsic proteins are bound to the lumenal side of PSII [13,14,15].

**Figure 1:**
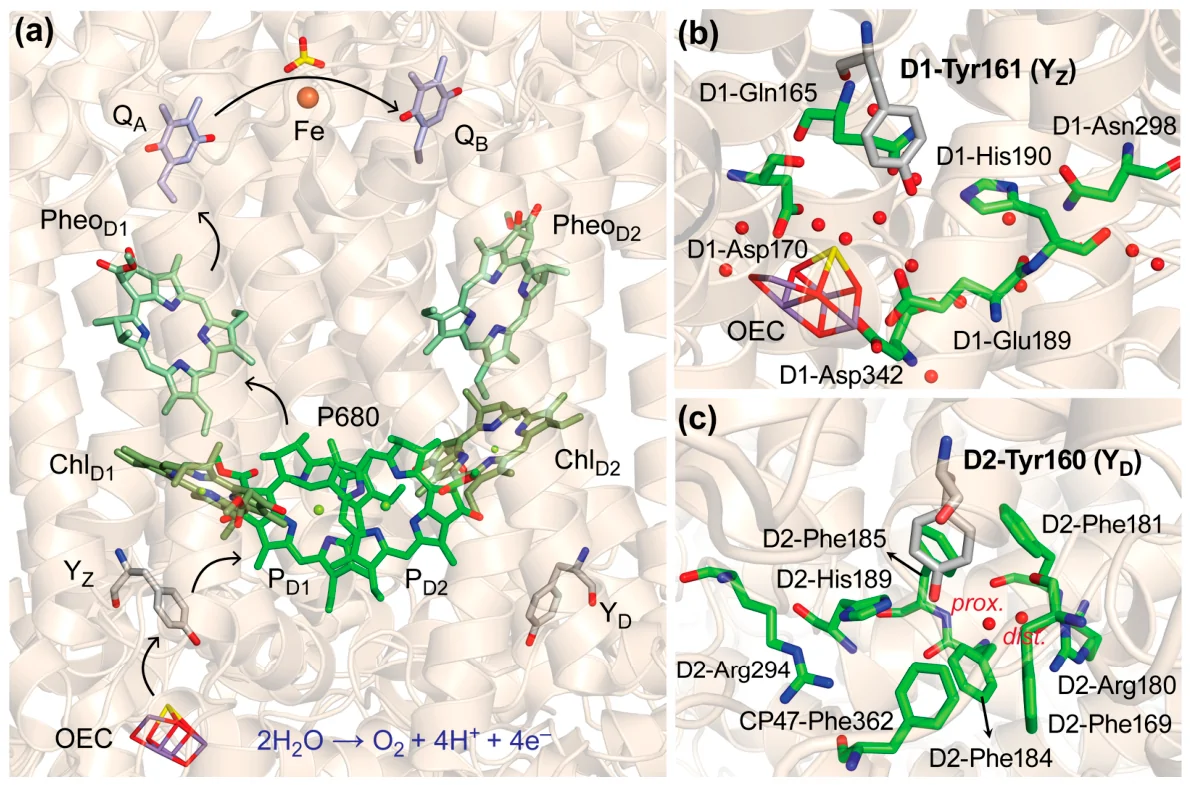
(**a**) Key redox-active cofactors involved in charge separation, electron transfer, and water oxidation in PSII. Curved arrows indicate the physiological electron-transfer direction from the OEC via Y_Z_ and the reaction-center pigments towards the quinone acceptors. (**b**,**c**) Comparison of the immediate environment of redox-active tyrosines Y_Z_ and Y_D_. Coordinates obtained from the 1.9 Å resolution crystallographic model of PSII with PDB ID 3WU2 [58].

The reaction center of PSII (Figure 1) contains four chlorophylls (Chls) and two pheophytins (Pheo) that are symmetrically positioned in two branches, D1 and D2. Excitation of the reaction center leads to fast charge separation within the D1 branch [8,16,17,18,19]. The primary photochemical event is likely associated with a charge-transfer state between Chl_D1_ and Pheo_D1_, which is specifically stabilized owing to the electrostatic anisotropy of the protein matrix [19,20,21,22]. Charge separation results in stabilization of the negative charge on Pheo_D1_ and of the positive charge on the central pair of chlorophylls, P_D1_ and P_D2_, within a few hundred fs [23,24]. As the primary electron acceptor, the redox potential for one-electron reduction of Pheo_D1_ dictates the driving force behind the initial charge separation and influences its quantum yield [25,26,27]. On the electron-acceptor side of PSII, charge separation is further stabilized by transferring the electron from Pheo^•−^ to plastoquinone (Q_A_) within ~300 ps [5]. The latter remains reduced for 0.5–10 ms before reducing, in turn, the terminal acceptor plastoquinone (Q_B_) [28,29,30]. This functions as a mobile electron carrier once it is reduced twice to plastoquinol and diffuses into the membrane [7].

The cationic radical formed on the P_D1_P_D2_ pair (commonly referred to using the spectroscopic term P680^•+^) is the strongest redox-cofactor oxidant in biology [3,31]. With an estimated reduction potential of ca. 1.2 V, it is able to drive the highly demanding oxidation of water to dioxygen [32]. This occurs in practice by stepwise oxidation of the OEC cluster with the help of an intermediate oxidant, redox-active D1-Tyr161 (Y_Z_) [9,33,34,35,36,37]. Oxidation of the OEC by Y_Z_^•^ advances the cluster through the cycle of catalytic states until O-O bond formation and O_2_ evolution are completed [38,39]. A second redox-active tyrosine, D2-Tyr160 (Y_D_), is positioned on the symmetry-related electron-transfer branch, which is not involved in the active water-oxidation pathway [40,41]. Although Y_D_ is not involved in the main electron-transfer (ET) pathway of PSII, it plays important regulatory roles that support the stable and efficient functioning of PSII [41,42,43,44,45,46,47]. Despite being spatially separated from the OEC by approximately 30 Å, Y_D_ has been associated with modulation of the OEC S-state catalytic cycle (S_0_-S_4_), indicating that its role extends beyond a passive auxiliary redox factor [42,43,48,49,50,51]. Y_D_ can be oxidized on the timescale of seconds by the S_2_ or S_3_ states of the OEC [43,51,52,53,54], while Y_D_^•^ may be reduced by the S_0_ state during dark adaptation [44,45], thereby helping the OEC return to the dark-stable S_1_ state. In addition, Y_D_ has been suggested to modulate electron transfer at the Y_Z_ site, possibly through electrostatic interaction with P680^•+^ [46,55,56,57].

Structurally, both redox-active tyrosines (Y_Z_ and Y_D_) are hydrogen-bonded to nearby histidine partners—D1-His190 for Y_Z_ and D2-His189 for Y_D_—but their second-sphere environments differ considerably (see Figure 1). Although both tyrosines are hydrogen-bonded to histidine partners, Y_Z_ is embedded in a water-rich hydrogen-bonding network involving D1-His190, D1-Asn298, D1-Glu189, D1-Asp342, D1-Asp170, D1-Gln165 and OEC-associated waters. In contrast, with the exception of D2-His189 and its hydrogen-bonding partner, D2-Arg294, Y_D_ is located in a comparatively hydrophobic phenylalanine-rich pocket [58,59,60,61,62,63,64] involving D2-Phe181, D2-Phe169, D2-Phe184, D2-Phe185, and CP47-Phe362. On the Y_D_ side, a microsolvating water molecule has been proposed to occupy two alternative positions (proximal and distal), where it can assist in oxidation-induced proton transfer from Y_D_ and thereby contribute to stabilization of the Y_D_^•^ [65,66]. These differences in hydration, polarity, hydrogen bonding, and local electrostatics provide a structural basis for the divergent redox potentials, radical lifetimes, and proton-coupled electron-transfer behavior of Y_Z_ and Y_D_ [65,66].

Prior studies suggested that the distinct kinetics exhibited by Y_Z_ and Y_D_ are influenced by their respective local hydrogen-bonding networks [57,65,66,67,68,69]. The hydrophilic local environment near Y_Z_ facilitates rapid oxidation and reduction, and the Y_Z_^•^ is short-lived, with reported decay half-lives of approximately 0.03–1 ms [70,71,72,73]. In contrast, the comparatively hydrophobic environment near Y_D_ accounts for its slower kinetics, and Y_D_^•^ is much longer-lived, decaying over minutes to hours [74]. These differences are accompanied by distinct estimated redox potentials, with Y_Z_ generally reported in the range of +900–1000 mV [43] and Y_D_ in the range of +700–800 mV [44,74].

More broadly, long-range biological electron transfer is not always limited to a direct donor–acceptor tunnelling event. In several redox proteins, such as class I ribonucleotide reductase [75,76,77,78,79,80,81,82], photolyase [83], ligninolytic and cytochrome-c peroxidases [84,85,86], ferredoxin-S-adenosylmethionine methyltransferase [87,88,89], and tetratricopeptide repeat proteins [90,91], the intervening protein matrix can influence electronic communication by positioning aromatic residues, particularly tyrosine and tryptophan, as possible relay or coupling elements. In PSII, the pronounced structural and kinetic asymmetry between Y_Z_ and Y_D_ raises a central mechanistic question: Does the intervening protein matrix merely organize the redox-active cofactors in space, or does it actively define branch-specific electron-hopping connectivity between the tyrosines and reaction-center chlorophylls of PSII?

Although PSII has a pseudo-symmetric D1/D2 architecture, charge separation and water oxidation proceed predominantly through the D1 branch, where Y_Z_ serves as the kinetically competent redox intermediate between the OEC and P680^•+^ [70,71,72,73,74]. By contrast, Y_D_ is positioned on the symmetry-related D2 branch and forms a comparatively stable radical [74], implying that the two tyrosines experience distinct electronic and electrostatic landscapes despite their structural analogy. Comparison of the connectivity between the redox-active tyrosines and the central chlorophyll pair (P_D1_P_D2_) will therefore provide a useful framework for examining whether the pseudo-symmetric protein scaffold preserves comparable geometric electron/hole-transfer connectivity on both branches or introduces branch-specific differences that may contribute to the divergent redox behavior. This is particularly relevant because the P_D1_P_D2_ chlorophyll pair is flanked by Y_Z_ and Y_D_ on opposite sides, and the intervening protein matrix contains aromatic residues whose possible contribution to electronic connectivity remains to be clarified.

Graph-based approaches offer a practical framework for examining long-range electron or hole transfer in large biomolecular systems, where full quantum-mechanical treatment of the entire protein is not feasible. In these models, residues and cofactors are treated as nodes, and possible communication routes are inferred from structural proximity, electronic coupling, and redox parameters. In the present work, eMap [92,93] was used to identify candidate electron/hole-transfer networks from the PSII architecture, while EHPath [94] provided a complementary kinetic ranking of donor–bridge–acceptor pathways using Marcus-type parameters and mean residence times. Although these methods do not provide directly experimentally comparable rate constants, they are well suited for comparing relative kinetic pathways in enzymes [95,96,97,98,99,100,101,102,103,104,105,106]. Using this combined strategy, we examined possible connections between Y_Z_/Y_D_ and the P_D1_P_D2_ chlorophylls. Our results reveal branch-specific differences in local aromatic networks: the Y_Z_–P_D1_ side is mainly associated with phenylalanine-assisted connectivity, whereas the Y_D_–P_D2_ side contains an additional tryptophan-associated route through D2-Trp191. Molecular dynamics (MD)-based conformational monitoring and residue conservation analysis were subsequently used to assess whether these proposed mediating residues remain structurally positioned and evolutionarily retained. Finally, multiscale quantum-mechanics/molecular-mechanics (QM/MM) calculations were used to probe the capacity of D2-Trp191 to accommodate an electron and thus act as ET mediator. Together, these results provide an enhanced and specific basis for understanding the distinct, branch-specific ET pathways in PSII.

## 2. Results and Discussion

The primary charge-separation events in PSII occur on ultrafast timescales and involve the reaction-center cofactors. Our focus here is instead on factors that affect subsequent ET events—specifically, on structural aspects that could be relevant for the oxidation of Y_Z_ and Y_D_ by the photochemically created electron hole in the P_D1_P_D2_ pair. To examine possible protein-matrix contributions to electronic connectivity in PSII, we focused on residues located between the redox-active tyrosines and the reaction-center chlorophyll pair, comparing the Y_Z_–P_D1_ and Y_D_–P_D2_ sides and analyzing possible matrix-associated connectivity. The identified pathways are subsequently used to suggest potential mediator candidates and branch-specific structural features.

### 2.1. Asymmetric Aromatic Features Between the Redox-Active Tyrosines and the Reaction Center

To assess whether the pseudo-symmetric protein scaffold of PSII contains comparable structural routes connecting the redox-active tyrosines with their immediate reaction-center chlorophylls, eMap pathway analysis was performed for the Y_Z_–P_D1_ and Y_D_–P_D2_ sides. This analysis is not intended to assign absolute electron-transfer rates but to identify low-penalty aromatic cofactor connectivity patterns between the donor-side tyrosines and the acceptor side P_D1_-P_D2_ chlorophyll region. In eMap analysis, each pathway score corresponds to the sum of distance-dependent edge-weight penalties along the route; therefore, lower scores indicate more compact and geometrically favorable connectivity within the graph model.

Although Y_Z_ is generally regarded as the kinetically competent electron donor to P680^•+^ during normal water oxidation, Y_D_ can also support rapid electron transfer under specific conditions, particularly at cryogenic temperature [107] and Mn-depleted PSII at alkaline pH, and has been proposed to contribute to photoassembly and photoprotection [46,55]. Against this functional background, on the Y_Z_ side, the lowest-score pathway connecting Y_Z_ with the P_D1_ chlorophyll was Y_Z_→D1-Phe186→P_D1_, with a score of 13.25 (Table 1). On the kinetically competent Y_Z_ side, the lowest-score pathway connecting Y_Z_ with the P_D1_ chlorophyll was Y_Z_→D1-Phe186→P_D1_, with a score of 13.25 (Table 1). A second low-penalty pathway, Y_Z_→D1-Phe182→P_D1_, was also identified, with a score of 15.44. These two routes indicate that the Y_Z_–P_D1_ side is dominated by phenylalanine-mediated connectivity, primarily through D1-Phe186 and D1-Phe182. Higher-score pathways involving additional cofactors (Figure S1) or more distant residues, such as nearby chlorophyll Chl_D1_, the oxygen-evolving complex (OEC401), or D1-Phe295, were not considered central to the present interpretation because their larger cumulative penalties reflect less favorable structural connectivity.

A closely analogous pattern was obtained on the Y_D_ side. The lowest-score pathway between Y_D_ and the P_D2_ chlorophyll was Y_D_→D2-Phe185→P_D2_, with a score of 13.23 (Table 2). This value is nearly identical to the lowest-score Y_Z_-side pathway, suggesting that the two pseudo-symmetric branches retain a comparable phenylalanine-mediated connection between the redox-active tyrosine and the adjacent reaction-center chlorophyll. Additional low-score pathways on the Y_D_ side include Y_D_→D2-Phe181→P_D2_, with a score of 15.18, and Y_D_→D2-Trp191→P_D2_, with a score of 15.37. The latter pathway is particularly noteworthy because it introduces a tryptophan-mediated route that is completely absent from the corresponding Y_Z_-side network.

Although the lowest-score pathways on both branches are phenylalanine-mediated, the electronic role of phenylalanine should be distinguished from that of tryptophan. Phenylalanine has an aromatic π-system and may support through-space electronic coupling, but it is relatively weak as an independent redox mediator because it is less able to stabilize radical/hole character than tyrosine or tryptophan. Thus, the phenylalanine-rich routes on the Y_Z_ side, particularly Y_Z_→D1-Phe186→P_D1_ and Y_Z_→D1-Phe182→P_D1_, are best interpreted as structurally favorable coupling pathways rather than discrete hole-hopping intermediates.

**Figure 2:**
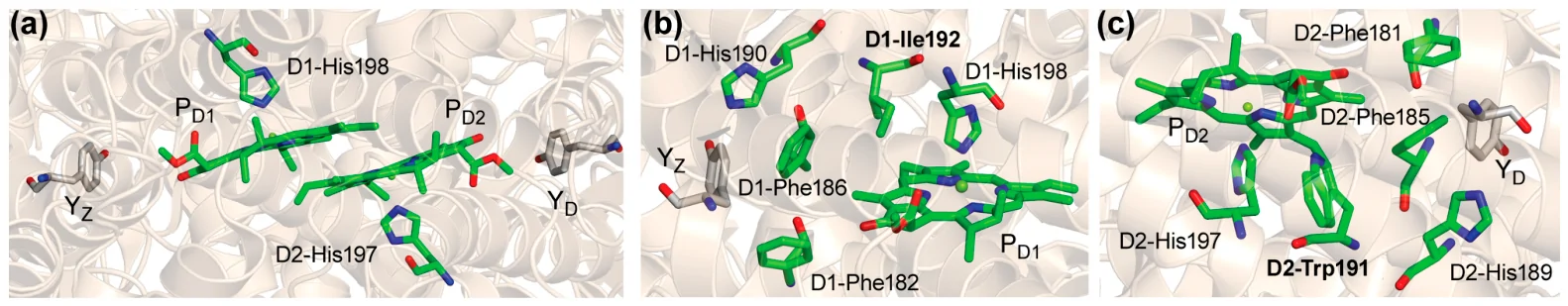
(**a**) Structural orientation of the Y_Z_–P_D1_ and Y_D_–P_D2_ branches in PSII. (**b**) Local Y_Z_–P_D1_ environment showing nearby residues. (**c**) Local Y_D_–P_D2_ environment showing nearby residues, including the branch-specific D2-Trp191.

In contrast, the Y_D_ side contains an additional low-penalty pathway involving D2-Trp191, Y_D_→D2-Trp191→P_D2_, with a score of 15.37. Although this score is slightly higher than the lowest phenylalanine-mediated route through D2-Phe185, D2-Trp191 is electronically more significant because tryptophan possesses an extended indole π-system, higher polarizability, and a greater ability to stabilize transient radical character. Therefore, the importance of D2-Trp191 may not be captured solely by its eMap score; rather, its chemical identity makes it a more plausible electron/hole-mediation site than phenylalanine. This distinction is noticeable in the branch-specific asymmetry observed in PSII. The symmetry-related position on the Y_Z_ side is occupied by D1-Ile190, an aliphatic residue lacking an aromatic π-system (Figure 2). Thus, PSII presents a potentially redox-relevant aromatic residue specifically in the Y_D_ branch. We interpret D2-Trp191 not as direct proof of a functional Y_D_→P_D2_ electron-transfer pathway but as a strategically positioned tryptophan that may tune the local electronic environment of the stable Y_D_^•^ site and deserves closer experimental and computational attention.

### 2.2. Comparative Screening of Electron-Hopping Pathways

Accurate theoretical prediction of charge-transfer kinetics in large biomolecular systems remains challenging because the rates depend on electronic coupling, local electrostatics, protonation state, dielectric response, and conformational dynamics. Accordingly, EHPath uses a reduced representation in which residues and cofactors are treated as redox nodes and transfer rates are estimated using Marcus-type distance-dependent expressions. For our study, EHPath electron-transfer screening (Tables S1 and S2) was used to evaluate possible electron-hopping routes between the redox-active tyrosines and the reaction-center chlorophylls. The calculated residence times are to be interpreted as comparative indicators of pathway accessibility rather than absolute physiological electron-transfer rates. This distinction is important because the functional difference between Y_Z_ and Y_D_ in PSII is influenced by proton-coupled electron transfer, hydrogen bonding, local electrostatics, and coupling to the OEC. Therefore, the purpose of this analysis is but to identify whether the two branches differ in their available electron-hopping architectures. The direct donor–chlorophyll pathways were found to occur on the same general timescale for both branches (Table 3). For the Y_Z_→P_D1_ side, the direct Y_Z_→P_D1_ pathway gave an exact residence time of 9.88 × 10^−7^ s (0.99 µs). Similarly, for the Y_D_→P_D2_ side, the direct Y_D_→P_D2_ pathway gave an exact residence time of 4.14 × 10^−7^ s (0.41 µs). These values suggest that both tyrosine–chlorophyll pairs are electronically connected in the EHPath model. Therefore, the main distinction between the two branches does not arise from the presence or absence of a direct pathway, but from the nature of the available mediated hopping routes.

For the Y_Z_→P_D1_ branch, the mediated pathways were weak compared with the direct route. When only phenylalanine residues were considered as bridge candidates, the best mediated pathway was Y_Z_→D1-Phe186→P_D1_, with an exact residence time of 8.92 s. The next pathway, Y_Z_→D1-Phe182→P_D1_, was even slower, with an exact residence time of 14.49 s. Thus, although phenylalanine residues are structurally present between Y_Z_ and P_D1_, they do not appear to provide an efficient electron-hopping relay in the EHPath screening. This supports the interpretation that the Y_Z_→P_D1_ branch is dominated by direct donor–chlorophyll electronic communication, with limited contribution from discrete phenylalanine-mediated hopping.

**Figure 3:**
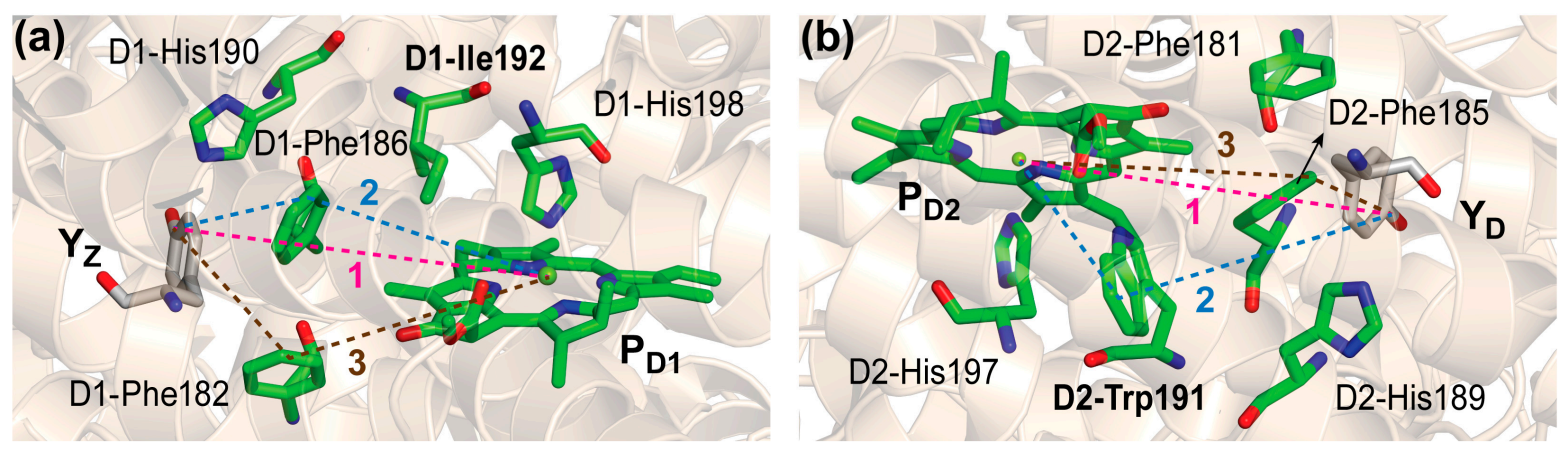
Structural representation of electron-hopping routes from redox-active tyrosines to reaction-center chlorophylls. (**a**) Possible pathways for Y_Z→_P_D1_ electron transfer; (**b**) possible pathways for Y_D→_P_D2_ electron transfer. Numbered dashed lines indicate representative EHPath routes, ranked from most favorable (1) to least favorable (3).

In contrast, the Y_D_→P_D2_ branch showed a distinct tryptophan-containing mediated pathway. In the neighboring matrix between donor and acceptor moieties, the most relevant mediated route was Y_D_→D2-Trp191→P_D2_, with an exact residence time of 2.50 × 10^−3^ s (2.50 ms). This pathway is slower than the direct Y_D_→P_D2_ pathway, as expected for a mediated hopping route (Figure 3). However, its significance lies in the identity of the bridging residue. D2-Trp191 contains an indole side chain, which is electronically more suitable for transient charge or radical delocalization than the phenyl ring of phenylalanine residues. Accordingly, the D2-Trp191-mediated pathway is much more favorable than the Phe-mediated alternatives on the same branch, such as Y_D_→D2-Phe185→P_D2_, which gives an exact residence time of 5.45 s. These results support the hypothesis that D2-Trp191 may serve as a transient electron-hopping site on the D2 side. The D2-Trp191 pathway should not be interpreted as a faster route than direct Y_D_→P_D2_ transfer. Rather, its slower residence time is consistent with a mediated hopping process in which electronic density can be transiently accommodated on the indole ring before further transfer toward P_D2_. The nearly identical approximate and exact residence times for Y_D_→D2-Trp191→P_D2_ further suggest that this route is not strongly penalized by backward hopping in the EHPath model. Therefore, D2-Trp191 is unlikely to be merely a passive structural residue; instead, it provides a chemically plausible electronic relay or stabilizing site near P_D2_.

Overall, the EHPath results indicate that both Y_Z_→P_D1_ and Y_D_→P_D2_ possess direct electronic connectivity on a comparable timescale. However, the mediated pathway analysis reveals a branch-specific difference: the Y_Z_→P_D1_ side lacks an efficient aromatic relay and is mainly direct, whereas the Y_D_→P_D2_ side contains D2-Trp191 as a plausible tryptophan-based hopping site. This supports a model in which D2-Trp191 contributes to electronic communication and redox stabilization on the D2 branch, potentially helping to tune the Y_D_–P_D2_ redox environment associated with the long-lived Y_D_^•^ state.

### 2.3. Dynamic Stability of Y_Z_/Y_D_ and Reaction-Center Connecting Residues

**Figure 4:**
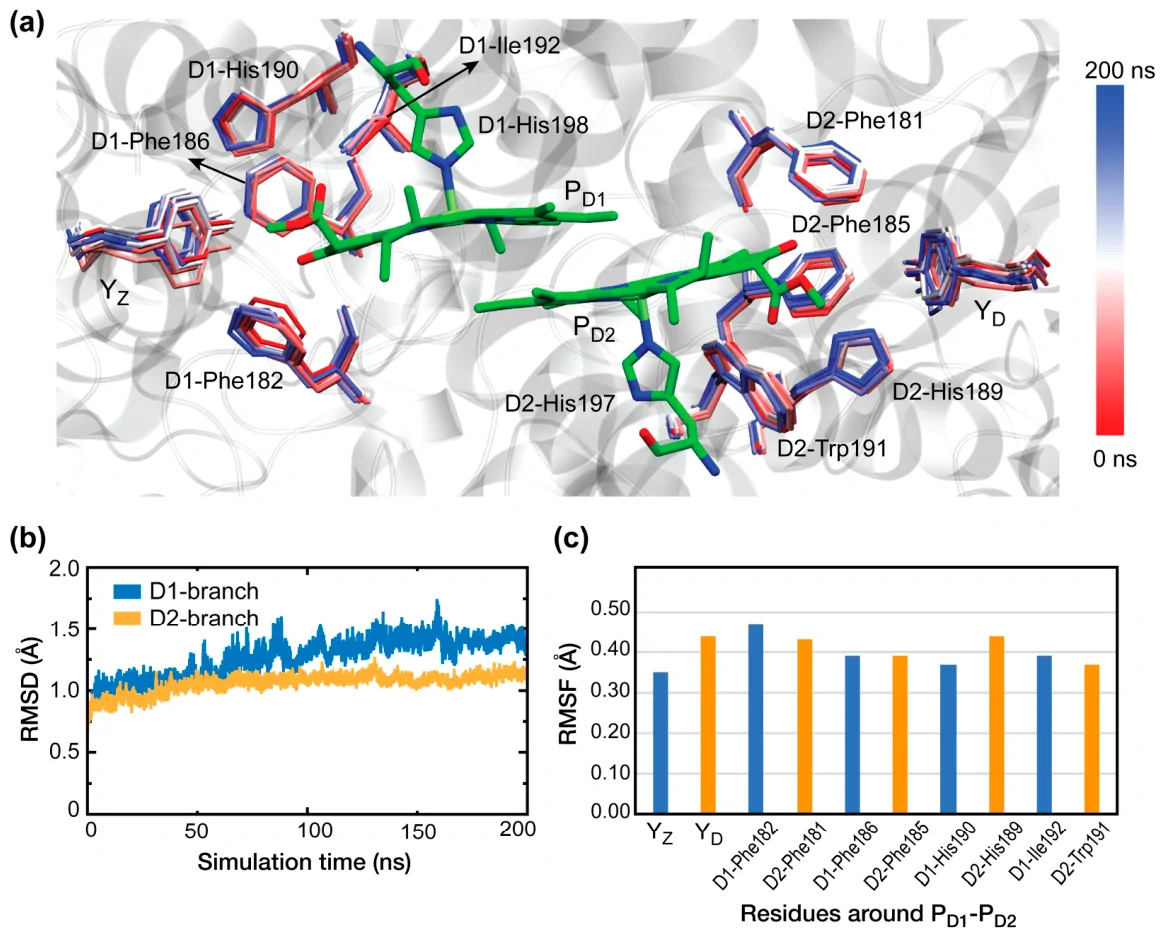
Dynamic stability of redox-active tyrosine environments in PSII. (**a**) Structural overlay of selected residues around the Y_Z_/P_D1_ and Y_D_/P_D2_ branches during the 200 ns MD simulation at a time interval of 20 ns. (**b**) RMSD profiles of the D1- and D2-branch residue sets. (**c**) Residue-wise RMSF values of key D1-branch residues plotted in blue bars and the corresponding spatially adjacent D2-branch residues plotted in orange bars, highlighting the structural stability of the tyrosine–chlorophyll interface.

To examine whether the residues identified in the electron-hopping analysis remain structurally positioned during protein dynamics, we analyzed a 200 ns MD trajectory of the PSII monomer model. Figure 4a shows representative structural overlays of the redox-active tyrosines, nearby aromatic residues and hydrogen-bonding residues along the trajectory. The residues surrounding both branches remain closely superimposed from 0 to 200 ns at each 20 ns, indicating that the local environments of Y_Z_ and Y_D_ are conformationally stable on the simulated timescale. This is further supported by the RMSD profiles of the D1- and D2-branch residue sets (see Figure 4b), which remain low throughout the simulation, suggesting that neither branch undergoes major rearrangement during the time evolution of MD simulation. In addition, distance profiles for the proposed donor–mediator–acceptor contacts in both D1 and D2 branches were calculated. These distances remained stable over the 200 ns trajectory, supporting the structural persistence of the proposed hopping geometries; the full distance plots, including the corresponding D2-branch analogous contacts, are provided in the Supporting Information (Figure S2 and Tables S3 and S4).

Residue-wise RMSF analysis also supports the rigidity of the local electron-hopping environment. On the D1 branch, the RMSF values of Y_Z_, D1-Phe182, D1-Phe186, D1-His190, and D1-Ile192 are 0.35, 0.47, 0.39, 0.37, and 0.39 Å, respectively, indicating very limited positional fluctuation. D1-Phe182 shows the highest fluctuation among the D1 residues, with an RMSF of 0.52 Å, but remains within a relatively low-fluctuation regime. On the D2 branch, Y_D_, D2-Phe181, D2-Phe185, D2-His189, and D2-Trp191 show RMSF values of 0.44, 0.43, 0.39, 0.44, and 0.37 Å, respectively. The low RMSF value of D2-Trp191 (0.37Å) is particularly important because it indicates that the tryptophan residue proposed as a possible electron-hopping site remains structurally rigid near the Y_D_/P_D2_ region during the 200 ns simulation. The comparison between the two branches therefore shows that the residues forming the local tyrosine–chlorophyll interface are not highly mobile or transiently positioned. Instead, both branches maintain a stable structural arrangement, with RMSF values mostly below 0.50 Å. In the context of the eMap and EHPath results, this further supports the interpretation that the D2-Trp191-mediated pathway is a persistent structural element of the D2-side protein matrix. The MD results therefore provide support for the proposed role of D2-Trp191 as a branch-specific residue capable of contributing to electronic communication or transient charge stabilization near P_D2_.

### 2.4. Electronic Character of the Proposed D2-Side Mediating Residues

QM/MM calculations were additionally set up and performed (Figure S3) to examine whether the selected mediating amino-acid residues can support the spin-accommodating character within the protein electrostatic environment. The QM region consisted of the mediating amino-acid residues identified from the pathway analysis, while the reaction-center chlorophyll acceptors, P_D1_P_D2_, were retained in the MM region. This partitioning allowed the electronic properties of the mediating residue network to be examined without artificially biasing the frontier orbitals toward the extended chlorophyll π-system while still retaining the electrostatic influence of the chlorophylls through the MM point charges. The D2 branch was selected as a case-study model because the EHPath analysis identified a possible tryptophan-containing route involving D2-Trp191. Tryptophan contains an extended π-electron system and can, in principle, participate in aromatic electron/hole-transfer mediation. In contrast, the spatially analogous position on the D1 side is occupied by D1-Ile192, which lacks an aromatic π-system. QM/MM geometry optimizations were performed using different combinations of possible donor and mediating residues, including Y_D_, D2-Phe181, D2-Phe185, D2-His189, and D2-Trp191, in the QM region in order to test whether specific aromatic residues can, in principle, accommodate unpaired-electron character within the protein environment.

**Figure 5:**
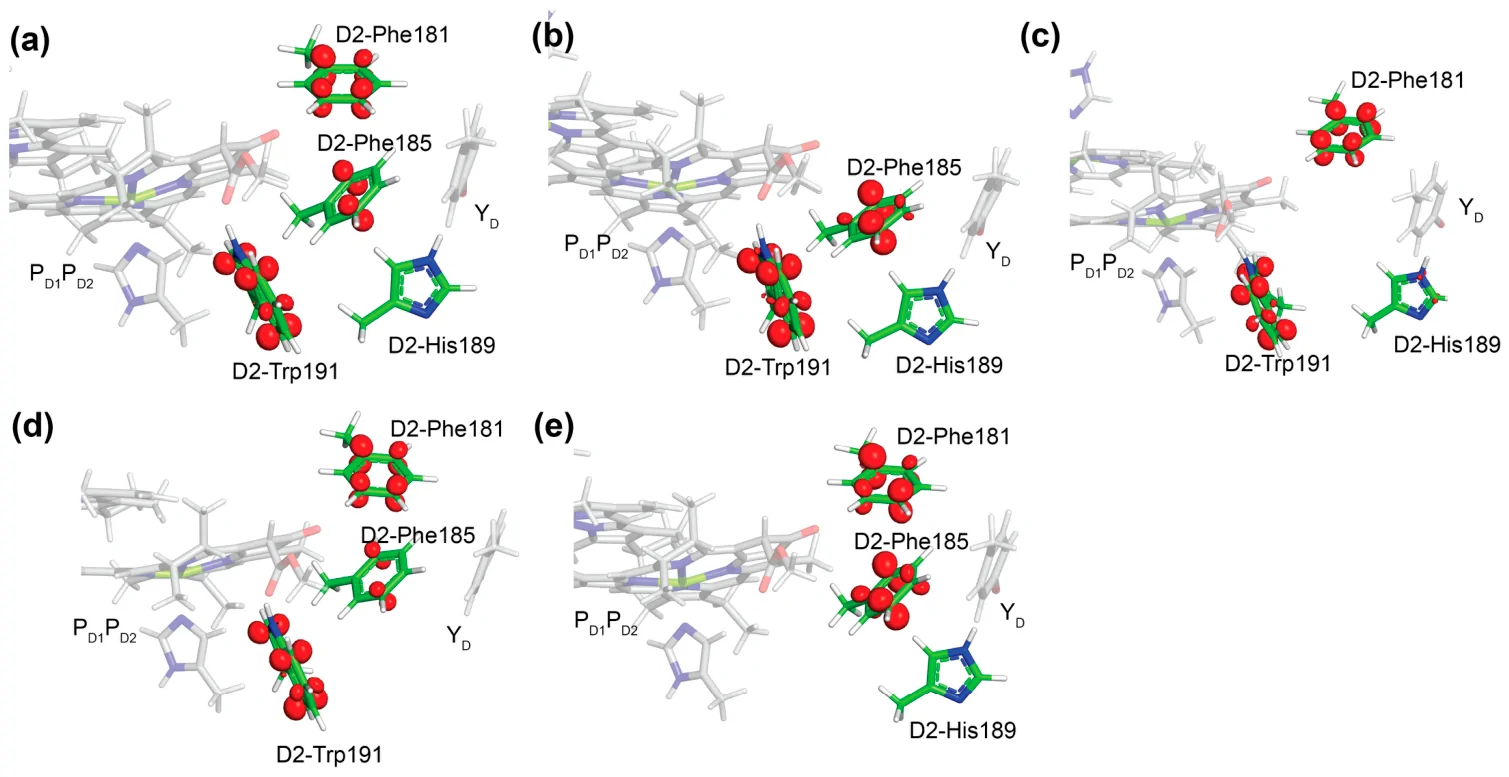
Depiction of spin-density distribution within the QM region of different QM/MM models used to probe radical localization along the proposed D2-side electron-transfer pathway. Panels (**a**–**e**) show different QM-region combinations of the proposed mediating residues, D2-Phe181, D2-Phe185, D2-Trp191, D2-His189, and Y_D_. Residues shown in green were included in the QM region. The reaction-center chlorophyll pair, P_D1_P_D2_, and Y_D_ are shown as semi-transparent in a truncated representation for clarity, but the full P_D1_P_D2_ cofactors were retained in the MM region and included through the electrostatic embedding scheme.

In the first set of calculations, where Y_D_ was included in the QM region, the spin density remained predominantly localized on Y_D_ (Figure S4). This is chemically reasonable, as formation of the stable Y_D_ radical is a well-established experimental feature of PSII [43,47,51,53,57,65,68]. Thus, the QM/MM result supports Y_D_ as the preferred radical-bearing site in the complete donor-containing model. To probe the intrinsic electronic response of the nearby mediating residues, additional models were generated in which Y_D_ was excluded from the QM region (Figure 5). In these reduced models, partial spin density was observed on D2-Phe181, D2-Phe185, and D2-Trp191, suggesting that these aromatic residues can, under constrained model conditions, accommodate some radical character. This observation is consistent with the availability of π-electron systems, which can provide possible transient mediation along a hopping pathway. Notably, no appreciable spin density was observed on D2-His189 in any model, consistent with its role as a redox-inactive hydrogen-bonding partner to Y_D_.

However, the spin population on D2-Phe181 and D2-Phe185 residues should be interpreted cautiously. Phenylalanine, in general, has a high redox potential (~2.0 V or higher) and is generally less redox-active than tyrosine (~0.94 V) or tryptophan (~1.05 V) [108,109]. Therefore, the apparent spin density may reflect local polarization or model-dependent delocalization rather than actual stability of a phenylalanine radical intermediate. In contrast, the involvement of D2-Trp191 is chemically plausible, and strong localization of spin on this residue is observed in all models where the tryptophan is included in the QM region. Overall, these QM/MM models provide qualitative support for possible D2-side aromatic mediation while confirming that Y_D_ remains the dominant stable radical site.

### 2.5. Evolutionary Conservation of the D1-Ile192/D2-Trp191 Asymmetry

To examine sequence-level conservation in the D1 and D2 reaction-center proteins in PSII, we compared the D1-PsbA and D2-PsbD gene sequences across representative PSII-containing organisms. The gene sequences were translated into their corresponding amino-acid sequences, allowing for residue-level alignment and comparison across organisms. This approach enabled us to map conserved and variable positions in the selected residue region and assess whether key residues near the PSII electron-transfer pathway are retained across different organisms.

**Figure 6:**
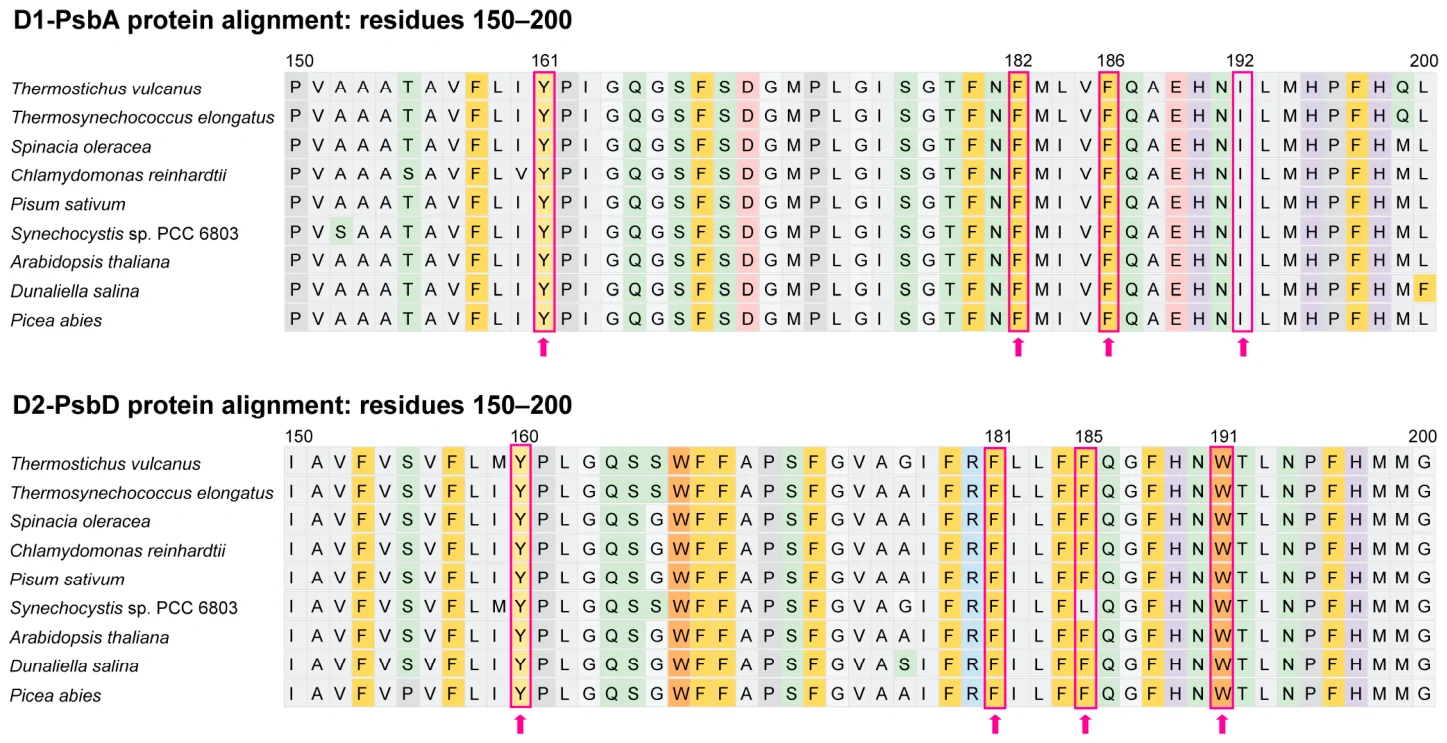
Sequence alignment of D1-PsbA and D2-PsbD residues in the residue range of 150–200 within various PSII structures and across different organisms. Residues closely positioned near the P_D1_P_D2_ chlorophyll pair are marked with arrows. Notice that D2-Phe185 is replaced by leucine in *Synechocystis* sp. 6803.

Figure 6 shows the aligned region spanning residues 150–200: this region contains the redox-active tyrosines and several neighboring residues positioned around the P_D1_P_D2_ chlorophyll interface. The alignment shows that the key D1/D2 asymmetry near the reaction-center chlorophylls is preserved across the examined organisms. Interestingly, in *Synechocystis* sp. PCC 6803, D2-Phe185 is naturally replaced by leucine. Although this substitution preserves the hydrophobic character of the local protein environment, leucine lacks the aromatic π-electron system of phenylalanine and is therefore unlikely to provide comparable electronic coupling. Nevertheless, spectroscopic comparisons between *Synechocystis* and spinach indicate no major differences in the Y_D_ environment [110]. Furthermore, the substantially longer mean residence time of the Phe185-mediated pathway (5.45 s) compared with the Trp-mediated pathway (2.50 × 10^−3^ s) supports the conserved Trp route as the dominant pathway. In the D1-PsbA branch, the position corresponding to D1-Ile192 remains occupied by an aliphatic residue, predominantly isoleucine, whereas the symmetry-related position in the D2-PsbD branch is strictly conserved as tryptophan corresponding to D2-Trp191 (Figure 6 and Figure S5). Thus, the D1-Ile192/D2-Trp191 asymmetry is an evolutionarily retained feature of the PSII reaction-center architecture rather than a species-specific structural residue.

Overall, we conclude that D2-Trp191 is not simply a structural residue but may act as an electronically competent aromatic element that modulates communication between Y_D_ and the reaction-center chlorophyll pair. In contrast to phenylalanine residues, which facilitate electronic coupling without efficiently stabilizing a hole, tryptophan can transiently accommodate radical character. Thus, D2-Trp191 may contribute both to rapid photooxidation of Y_D_, as evidenced under specific conditions [46,55,107], and to the distinctive stability of Y_D_^•^. This hypothesis does not imply an obligatory Y_D_ electron-transfer pathway but proposes that D2-Trp191 serves as local mediator within the D2 branch.

## 3. Conclusions and Outlook

In this work, we examined whether the pseudo-symmetric D1 and D2 branches of PSII provide equivalent or distinct electron/hole-transfer connectivity between the redox-active tyrosines and the reaction-center chlorophylls. The combined eMap and EHPath analyses show that both Y_Z_–P_D1_ and Y_D_–P_D2_ possess direct electronic connectivity, but their mediated pathways are chemically distinct. The Y_Z_/P_D1_ branch is dominated by phenylalanine-mediated structural coupling, whereas the Y_D_/P_D2_ branch contains D2-Trp191 as a uniquely positioned residue capable of providing stronger electronic tuning than the corresponding phenylalanine-mediated alternatives. MD simulations further show that D2-Trp191 remains conformationally stable, indicating that this feature is not a transient structural arrangement but a persistent part of the D2-side redox environment. QM/MM calculations further support this interpretation by showing appreciable spin localization on D2-Trp191 within the proposed D2-side aromatic pathway, suggesting its capacity to accommodate transient radical character within the local protein electrostatic environment. Sequence comparison across representative PSII structures from cyanobacteria, green algae, higher plants, and gymnosperms further supports the conservation of the D1-Ile192 and D2-Trp191 asymmetry near the reaction-center chlorophyll pair. Together, these results suggest that the PSII protein matrix does more than maintain cofactor geometry; it imposes branch-specific electronic communication asymmetry that may help tune the distinct redox behaviors and ET kinetics of Y_Z_ and Y_D_. This study therefore provides a structural and computational basis for better understanding how conserved protein-matrix features contribute to redox specialization within the PSII reaction center.

## 4. Materials and Methods

### 4.1. eMap-Based Identification of Possible Connectivity Pathways

Electron/hole-transfer connectivity between the redox-active tyrosines and the reaction-center chlorophylls was examined using eMap 2.0 [92], a graph-based tool for identifying possible electron or hole-transfer pathways in protein structures. eMap represents aromatic amino-acid side chains and non-protein electron-transfer-active cofactors as nodes in a distance weighted graph and calculates possible pathways using distance-dependent edge weights based on a coarse-grained version of the Beratan–Onuchic pathway model [111,112]. A cleaned monomer unit of the PSII structural model was used as the input PDB file (PDB ID: 3WU2) [58]. The analysis was performed separately for the two pseudo-symmetric branches: Y_Z_ to the P_D1_ chlorophyll and Y_D_ to the P_D2_ chlorophyll. Aromatic residues and automatically identified non-protein ET-active moieties (other organic and inorganic cofactors), including chlorophyll fragments, were retained for graph construction. Pairwise distances between selected residues/co-factors were used to generate the weighted graph, where each edge weight was calculated as a modified distance-dependent hopping penalty. The total pathway score was defined as the sum of edge weights along a given pathway; therefore, lower scores indicate lower-penalty structural routes, not absolute electron-transfer rates. For each tyrosine–chlorophyll pair, source–target pathway searches were performed to identify the shortest candidate routes. The resulting pathways were ranked by score and inspected to identify recurring aromatic mediators, with particular attention to Phe, His, and Trp residues. Pathways involving axial histidine ligands were interpreted cautiously because their inclusion may reflect direct chlorophyll coordination rather than independent hopping steps.

### 4.2. EHPath-Based Theoretical Kinetic Screening of Electron/Hole-Transfer Pathways

Electron/hole-transfer pathways between the redox-active tyrosines and the reaction-center chlorophylls were evaluated using EHPath [94], a graph-based kinetic tool that ranks possible hopping pathways according to the mean residence time (*τ*_M_) of the transferring charge. EHPath estimates individual charge-transfer rates using a Marcus-type expression, then combines these rates to obtain approximate and exact residence times for each donor–bridge–acceptor pathway [95]. The method was originally developed to identify electron-/hole-hopping routes in proteins and nucleic acids, particularly through redox-active amino-acid residues and cofactors [94]. For the present PSII analysis, Y_Z_ and Y_D_ were treated as donor nodes, while reaction-center chlorophylls P_D1_ and P_D2_ were treated as acceptor nodes. Candidate bridging residues were selected based on structural visual inspection around the donor and acceptor, and potential residues were identified by eMap analysis, with particular focus on redox-active residues. The input files were prepared as donor, bridging, and acceptor CSV files containing atom names, residue names, residue numbers, chain identifiers, and Cartesian coordinates.

EHPath requires residue/cofactor-specific Marcus parameters: the reduction potential (E), reorganization-energy parameter (λ), effective radius (r), and donor–bridge–acceptor coordinates. The values used for Tyr and Trp were retained from the EHPath script: λ= 1.02 eV and E = 0.93 V for Tyr and λ = 0.95 eV and E = 1.02 V for Trp [94]. These values are consistent with the recognized roles of Tyr and Trp as biologically relevant hole-hopping residues and with reported reduction potentials of tyrosyl and tryptophanyl radicals near physiological pH [109]. For chlorophyll acceptor nodes, λ = 0.315 eV was assigned as one-half of the reported vibrationally averaged hole reorganization energy (required by the EHpath algorithm) of chlorophyll, while E = 1.20 V was used as an effective PSII reaction-center chlorophyll potential reflecting the high oxidizing character of the P680^•+^ environment [31,113]. Thus, the chlorophyll parameters combine a pigment-specific reorganization term with a protein-tuned potential and were used for comparative pathway ranking rather than absolute kinetic prediction. For phenylalanine, the reduction potential was assigned based on literature reporting phenylalanine [114]. Histidine was included only where relevant as a chemically plausible but environment-sensitive redox/electrostatic residue, given the known sensitivity of imidazole oxidation and the protonation state to the local environment [115]. Since the same framework and parameter set were applied consistently across all pathways, the mean residence times calculated by EHpath provide a useful basis for relative comparison for pathways rather than absolute kinetic prediction.

### 4.3. Molecular Dynamics Simulations

The molecular dynamics trajectory analyzed in the present work was taken from previously reported simulations performed in our group [20,21,22,66,116]. The complete details of the preparation of the PSII system, cofactor parametrization, membrane embedding, solvation, ion placement, energy minimization, gradual heating, density equilibration, equilibration, and MD production protocol were described in the work of Sirohiwal and Pantazis [116]. Therefore, only the trajectory analysis relevant to the present study is described here. Briefly, the 200 ns production trajectory was analyzed to monitor the structural stability of residues located between the redox-active tyrosines and the reaction-center chlorophylls, with particular attention to the (Y_Z_/P_D1_) and (Y_D_/P_D2_) interfacial regions. Root mean square deviation (RMSD) and residue-wise root mean square fluctuation (RMSF) analyses were performed for selected D1- and D2-branch residues to evaluate whether the proposed electron-hopping residues remained geometrically stable during the simulation.

### 4.4. QM/MM Calculations

QM/MM calculations were performed to examine whether the proposed mediating residues can support localized electronic character in the protein environment. A representative structure was selected from the MD trajectory by clustering based on Cα-atom deviations of the protein matrix. Since the region of interest is buried within the PSII protein core and is spatially separated from the membrane-facing lipid environment, lipid molecules were removed before QM/MM model preparation. To retain the local electrostatics and hydrogen-bonding environment of the protein, approximately 8000 water molecules surrounding the PSII core region were kept. Mobile ions were removed from the truncated QM/MM model (Figure S3) to avoid artificial long-range electrostatic effects from incompletely solvated or boundary-proximal ions after system reduction. The QM region contained the donor and mediating amino-acid residues of the selected D2-side pathway. The P_D1_P_D2_ acceptor pair was placed in the MM region so that its electrostatic influence was included through point charges without forcing the frontier orbitals to delocalize onto the extended chlorophyll π-system. The active region for QM/MM geometry optimization was defined as the protein residues and water molecules located within 8 Å of the centrally positioned D2-Phe185 residue in the QM region. Atoms outside this active region were kept fixed and represented as MM point charges. All QM/MM calculations were carried out using the quantum chemistry package ORCA 6.0 [117]. The MM region was described using the ORCA force-field framework. Electrostatic embedding was used so that the QM electron density was polarized by the surrounding MM point charges. Covalent bonds crossing the QM/MM boundary were treated using the link-atom approach, and charge redistribution at the boundary was handled using the charge-shift model. The QM region was optimized using the PBE [118] functional with the def2-SVP basis set [119], including dispersion corrections [120]. A doublet spin state was used to represent the one-electron oxidized donor state of Y_D_. This allows the distribution of the singly occupied orbital, associated with Y_D_^•^ and mediator radical character, to be examined within the local protein environment.

## Figures and Tables

**Table 1:** eMap-predicted pathways from Y_Z_- to the P_D1_-side chlorophyll. The arrow (→) indicates the predicted direction of electron transfer.

Path ID	Predicted Pathway	Score	Edge Weight
1a	Y_Z_→D1-Phe186→P_D1_	13.25	4.51, 8.74
1b	Y_Z_→D1-Phe182→P_D1_	15.44	6.05, 9.39
1c	Y_Z_→D1-Phe186→D1-Phe182→P_D1_	19.36	4.51, 5.46, 9.39
1d	Y_Z_→D1-Phe182→D1-Phe186→P_D1_	20.24	6.05, 5.46, 8.74
1e	Y_Z_→D1-Phe182→Chl_D1_→P_D1_	26.34	6.05, 10.75, 9.54
1f	Y_Z_→OEC401→D1-Phe186→P_D1_	27.63	8.23, 10.66, 8.74
1g	Y_Z_→OEC401→D1-Phe182→P_D1_	28.22	8.23, 10.60, 9.39
1h	Y_Z_→D1-Phe295→D1-Phe186→P_D1_	29.61	9.25, 11.62, 8.74
1i	Y_Z_→D1-Phe186→OEC401→D1-Phe182→P_D1_	35.16	4.51, 10.66, 10.60, 9.39
1j	Y_Z_→D1-Phe295→D1-Phe186→D1-Phe182→P_D1_	35.72	9.25, 11.62, 5.46, 9.39

**Table 2:** eMap-predicted pathways from Y_D_- to the P_D2_-side chlorophyll. The arrow (→) indicates the predicted direction of electron transfer.

Path ID	Predicted Pathway	Score	Edge Weight
1a	Y_D_→D2-Phe185→P_D2_	13.23	4.60, 8.63
1b	Y_D_→D2-Phe181→P_D2_	15.18	5.86, 9.32
1c	Y_D_→D2-Trp191→P_D2_	15.37	9.19, 6.18
1d	Y_D_→D2-Phe185→D2-Trp191→P_D2_	16.68	4.60, 5.89, 6.18
1e	Y_D_→D2-Phe185→D2-Phe181→P_D2_	18.26	4.60, 4.34, 9.32
1f	Y_D_→D2-Phe181→D2-Phe185→P_D2_	18.83	5.86, 4.34, 8.63
1g	Y_D_→D2-Phe181→D2-Trp191→P_D2_	21.16	5.86, 9.12, 6.18
1h	Y_D_→D2-Phe184→D2-Phe185→P_D2_	22.12	7.05, 6.44, 8.63
1i	Y_D_→D2-Phe184→D2-Phe181→P_D2_	23.79	7.05, 7.42, 9.32
1j	Y_D_→D2-Trp191→D2-Phe180→P_D2_	30.36	9.19, 10.50, 10.67

**Table 3:** Screening of Y_Z_/Y_D_-to-P_D1_P_D2_ electron-hopping routes in PSII. The arrow (→) indicated the direction of electron transfer.

Branch	Pathway Category	Pathway	EHPath Predicted Mean Residence Time (*τ*_M_) in Sec.
Y_Z_→P_D1_	Direct	Y_Z_→P_D1_	9.88 × 10^−7^
Y_Z_→P_D1_	1st mediated	Y_Z_→D1-Phe186→P_D1_	8.92
Y_Z_→P_D1_	2nd mediated	Y_Z_→D1-Phe182→P_D1_	14.49
Y_D_→P_D2_	Direct	Y_D_→P_D2_	4.14 × 10^−7^
Y_D_→P_D2_	1st mediated	Y_D_→D2-Trp191→P_D2_	2.50 × 10^−3^
Y_D_→P_D2_	2nd mediated	Y_D_→D2-Phe185→P_D2_	5.45

## Data Availability

The data that support the findings of this study are available in the Supplementary Materials of this article. Electronic Supplementary Information (ESI) available: Figures S1–S4 and Tables S1–S5.

## Supplementary Materials

The following supporting information can be downloaded at: https://www.mdpi.com/article/10.3390/plants15172557/s1, Table S1: EHPath electron-transfer screening for Y_Z_→P_D1_ using neighboring potential amino-acid residues; Table S2: EHPath electron-transfer screening for Y_D_→P_D2_ using neighboring potential amino-acid residues; Table S3: Root mean square fluctuation values for the potential ET-mediating residues between Y_Z_-P_D1_ and Y_D_-P_D2_; Table S4: Average donor–mediator and donor–acceptor distances for proposed electron transfer contacts in D1 and D2 units of PSII; Table S5: Representative PSII structures from cyanobacteria, green algae, higher plants, and gymnosperms used for comparative structural and positional-conservation analysis, including organism source, experimental method, PDB accession, and reported resolution; Figure S1: Structural organization and predicted electron-transfer pathway networks in Photosystem II; Figure S2: Distance stability of proposed tyrosine–chlorophyll electron/hole-transfer contacts during the 200 ns MD trajectory; Figure S3: QM/MM model and active region used for the PSII donor–mediator calculation; Figure S4: Spin distribution within models, with the donor site considered in the QM region.; Figure S5: Positional conservation of key D1 and D2 residues across different PSII organisms based on superimposed structural units (PDB IDs: 3WU2 [58], 1W5C [121], 3JCU [121], 6KAC [121], 6YP7 [122], 7N8O [123], 7OUI [124], 7PI0 [124], and 8C29 [125]).
