# Supplementary material for: Asymmetric Connectivity Between Redox-Active Tyrosines and Reaction-Center Chlorophylls in Photosystem II
Source: Plants (Basel). 2026 Aug 22;15(17):2557. doi: 10.3390/plants15172557 (PMC13567741; doi:10.3390/plants15172557)
Supplement: Supplementary file 1 [file plants-15-02557-s001.zip › plants-4439034-supplementary.pdf]

Supporting Information

Asymmetric Connectivity Between Redox-Active Tyrosines and Reaction-Center Chlorophylls in Photosystem II

Shalini Yadav, Dimitrios A. Pantazis

Table of Contents

**Table S1**	EHPath electron-transfer screening for $Y_Z \rightarrow P_{D1}$	Page 2
**Table S2**	EHPath electron-transfer screening for $Y_D \rightarrow P_{D2}$	Page 2
**Figure S1**	Structural organization and predicted ET pathways from Y_Z/Y_D to P_{D1}/P_{D2}	Page 3
**Table S3**	Root mean square fluctuation values for the potential ET mediating residues between Y_Z-P_{D1} and Y_D-P_{D2}	Page 4
**Table S4**	MD average distance plot between residues involved in Y_Z/Y_D to $P_{D1}P_{D2}$ ET	Page 4
**Figure S2**	Distance stability of proposed tyrosine–chlorophyll electron/hole-transfer contacts during the 200 ns MD trajectory	Page 5
**Figure S3**	QM/MM model and active region used for the PSII donor-mediator calculation	Page 5
**Figure S4**	Spin distribution when Y_D site was considered in the QM region	Page 6
**Table S5**	Representative PSII structures from different organisms for positional comparison of key residues	Page 6
**Figure S5**	Positional conservation of D1-Ile192 and D2-Trp191 across different organisms	Page 7

Table S1. EHPath electron-transfer screening for $Y_Z \rightarrow P_{D1}$ using neighboring potential amino acid residues.

Rank	Pathway assignment	Mean residence time (τ_M)	Readable time
1	$Y_Z \rightarrow P_{D1}$	9.88×10^{-7} s	0.99 μs
2	$Y_Z \rightarrow$ D1-Phe186 $\rightarrow P_{D1}$	8.92×10^{0} s	8.92 s
3	$Y_Z \rightarrow$ D1-Phe182 $\rightarrow P_{D1}$	1.45×10^{0} s	14.49 s
4	$Y_Z \rightarrow$ D1-Phe182 $\rightarrow$ D1-Phe186 $\rightarrow P_{D1}$	1.78×10^{1} s	17.83 s
5	$Y_Z \rightarrow$ D1-Phe186 $\rightarrow$ D1-Phe182 $\rightarrow P_{D1}$	2.90×10^{1} s	28.99 s

Table S2. EHPath electron-transfer screening for $Y_D \rightarrow P_{D2}$ using neighboring potential amino acid residues.

Rank	Pathway assignment	Mean residence time (τ_M)	Readable time
1	$Y_D \rightarrow P_{D2}$	4.14×10^{-7} s	0.41 μs
2	$Y_D \rightarrow$ D2-Trp191 $\rightarrow P_{D2}$	2.50×10^{-3} s	2.50 ms
3	$Y_D \rightarrow$ D2-Phe185 $\rightarrow P_{D2}$	5.45×10^{0} s	5.45 s
4	$Y_D \rightarrow$ D2-Trp191 $\rightarrow$ D2-Phe185 $\rightarrow P_{D2}$	5.45×10^{0} s	5.45 s
5	$Y_D \rightarrow$ D2-Phe181 $\rightarrow$ D2-Phe185 $\rightarrow P_{D2}$	1.09×10^{1} s	10.89 s

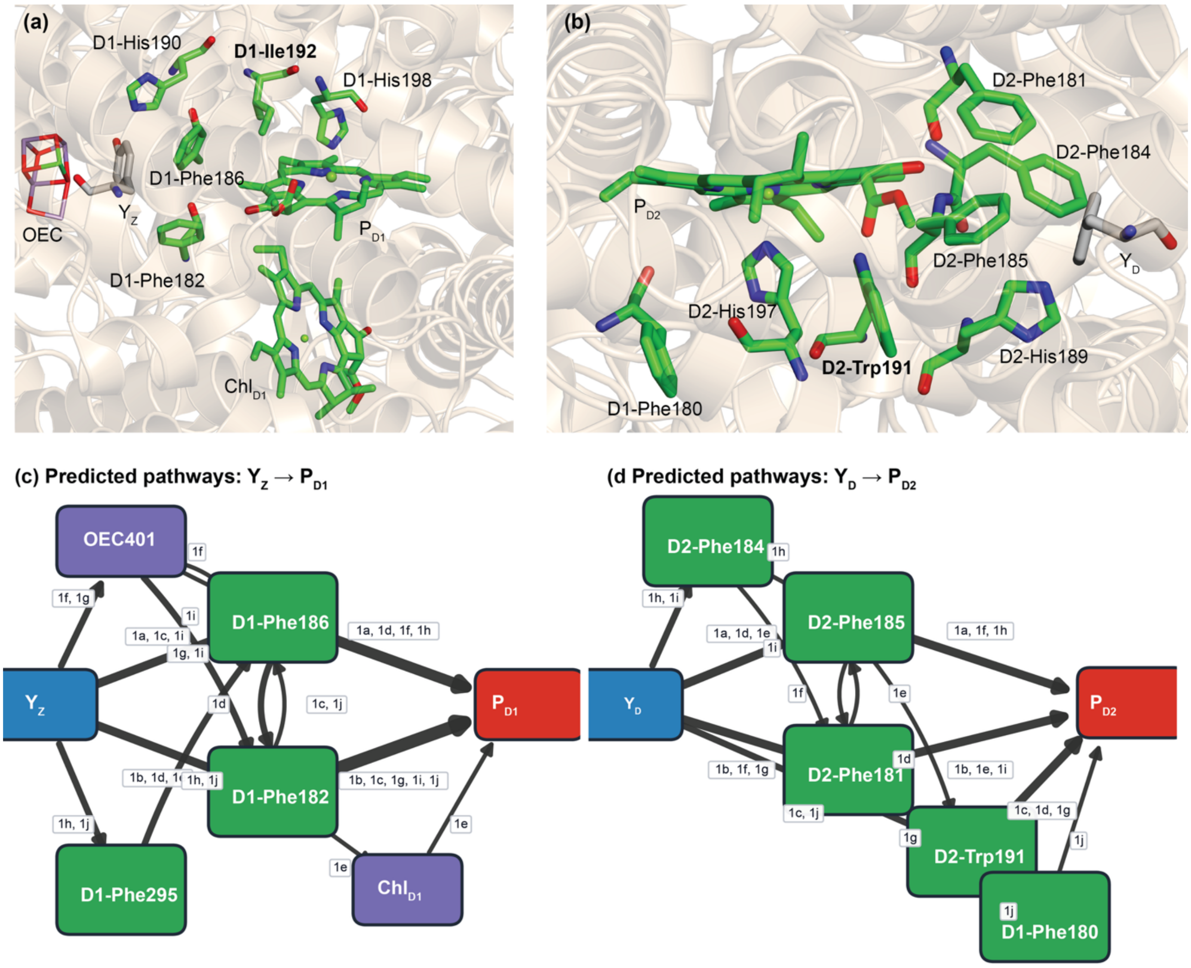

Figure S1. Structural organization and predicted electron-transfer pathway networks in Photosystem II. (a, b) Spatial arrangement of the redox-active cofactors and surrounding aromatic residues associated with the D1-side $Y_Z \rightarrow P_{D1}$ and D2-side $Y_D \rightarrow P_{D2}$ pathways, respectively. (c, d) Combined predicted pathways from $Y_Z \rightarrow P_{D1}$ and from $Y_D \rightarrow P_{D2}$. Blue, green, purple, and red nodes represent the initial tyrosine donor, intervening aromatic residues, cofactors, and terminal reaction center chlorophyll acceptor, respectively. Arrows indicate the predicted direction of transfer, while the accompanying labels identify the corresponding path IDs listed in tables S1 and S2. Connection thickness reflects the frequency with which a given transfer step occurs among the predicted pathways, with thicker connections representing more frequently shared steps.

Table S3. Root mean square fluctuation values for the potential ET mediating residues between Y_Z-P_{D1} and Y_D-P_{D2}.

Residues	RMSF (Å)
D1-Tyr161 (Y_Z)	0.35
D2- Tyr160 (Y_D)	0.44
D1-Phe182	0.47
D2-Phe181	0.43
D1-Phe186	0.39
D2-Phe185	0.39
D1-His190	0.37
D2-His189	0.44
D1-Ile192	0.39
D2-Trp191	0.37

Table S4. Average donor–mediator and donor–acceptor distances for proposed electron transfer contacts in D1 and D2 units of PSII.

Residue 1	Residue 2	Distance (Å)	Residue 1	Residue 2	Distance (Å)
Y_Z	P_{D1}:MG	14.28	Y_D	P_{D2}:MG	14.28
Y_Z	D1-Phe182	7.77	Y_D	D2-Phe181	8.46
Y_Z	D1-Phe186	6.78	Y_D	D2-Phe185	8.07
Y_Z	D1-His190	8.26	Y_D	D2-His189	9.01
Y_Z	D1-Ile192	11.44	Y_D	D2-Trp191	10.99

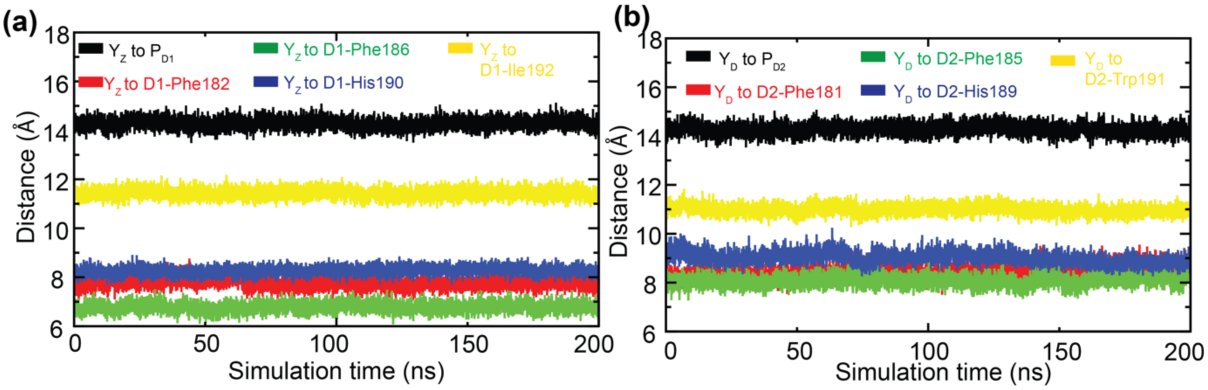

Figure S2. Distance stability of proposed tyrosine–chlorophyll electron/hole-transfer contacts during the 200 ns MD trajectory. (a) Distance profiles for selected Y_Z–P_{D1} and Y_Z-associated D1-branch contacts. (b) Distance profiles for selected Y_D–P_{D2} and Y_D-associated D2-branch contacts, including the proposed D2-Trp191-mediated route. The largely stable distance traces indicate that the proposed donor–mediator–acceptor geometries remain well maintained during the simulation.

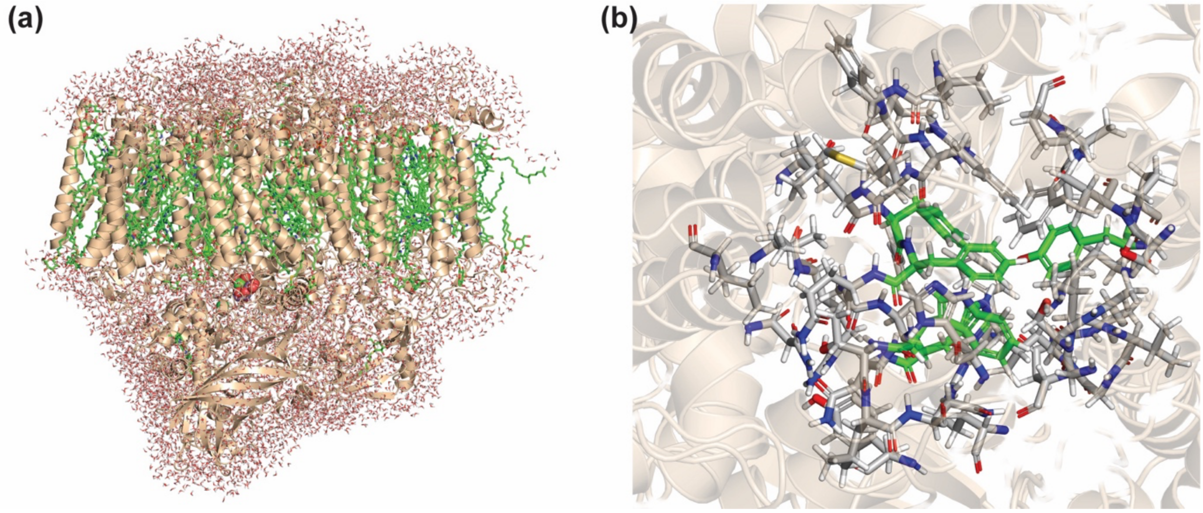

Figure S3. QM/MM model and active region used for the PSII donor-mediator calculation. (a) Truncated PSII QM/MM model after removal of the lipid environment, retaining the protein core and surrounding water molecules. (b) Enlarged view of the QM/MM active region containing 588 atoms around the proposed D2-side donor–mediator pathway. The QM region containing the donor and mediating residues is shown in green, while nearby active-region residues treated at the MM level are shown in gray.

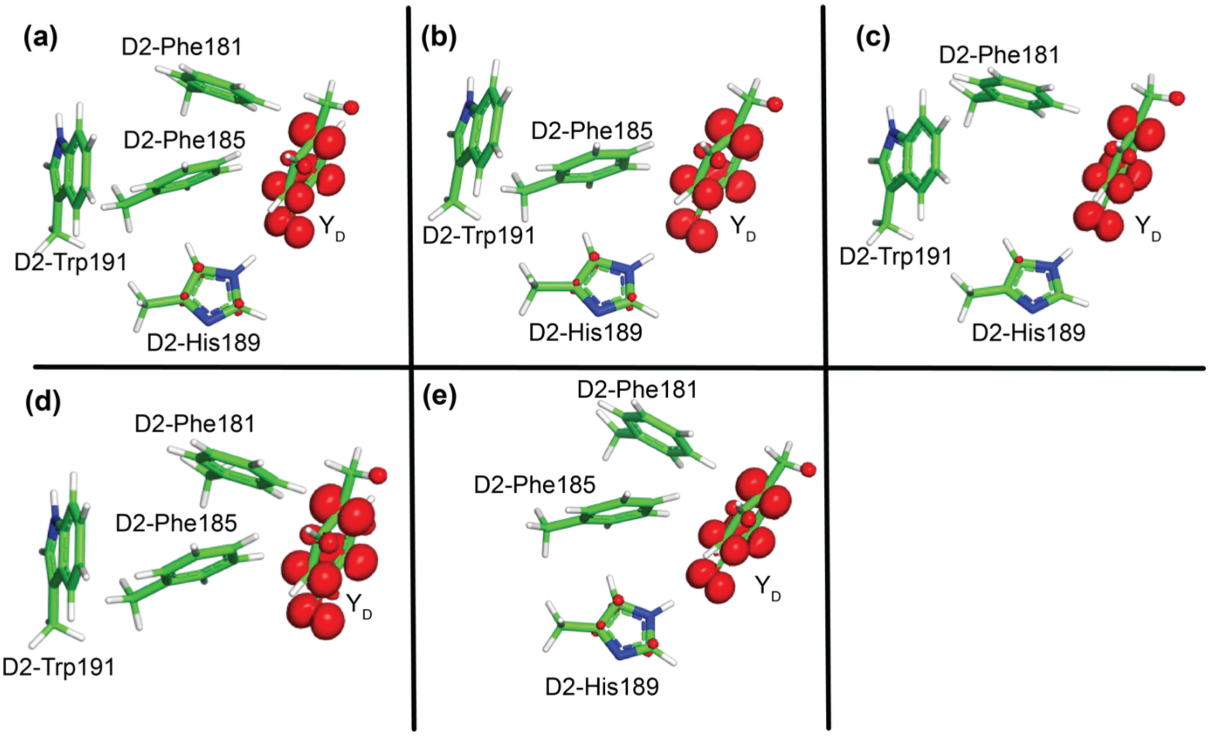

Figure S4. Spin distribution within models, with the donor site considered in the QM region. Note that in this case the spin density is mostly localized on the donor site.

Table S5. Representative PSII structures from cyanobacteria, green algae, higher plants, and gymnosperms used for comparative structural and positional-conservation analysis, including organism source, experimental method, PDB accession, and reported resolution.

**Organism**	**Group**	**PDB ID**	**Method**	**Resolution (Å)**
Thermostichus vulcanus	Thermophilic cyanobacterium	3WU2 [1]	XRD	1.90
Thermosynechococcus elongatus	Thermophilic cyanobacterium	1W5C [2]	XRD	3.20
Spinacia oleracea	Higher plant	3JCU [2]	Cryo-EM	3.20
Chlamydomonas reinhardtii	Green alga	6KAC [2]	Cryo-EM	2.70
Pisum sativum	Higher plant	6YP7 [3]	Cryo-EM	3.80
Synechocystis sp. PCC 6803	Mesophilic cyanobacterium	7N8O [4]	Cryo-EM	1.93
Arabidopsis thaliana	Higher plant	7OUI [5]	Cryo-EM	2.79
Dunaliella salina	Green alga	7PI0 [5]	Cryo-EM	2.43
Picea abies	Gymnosperm	8C29 [6]	Cryo-EM	2.80

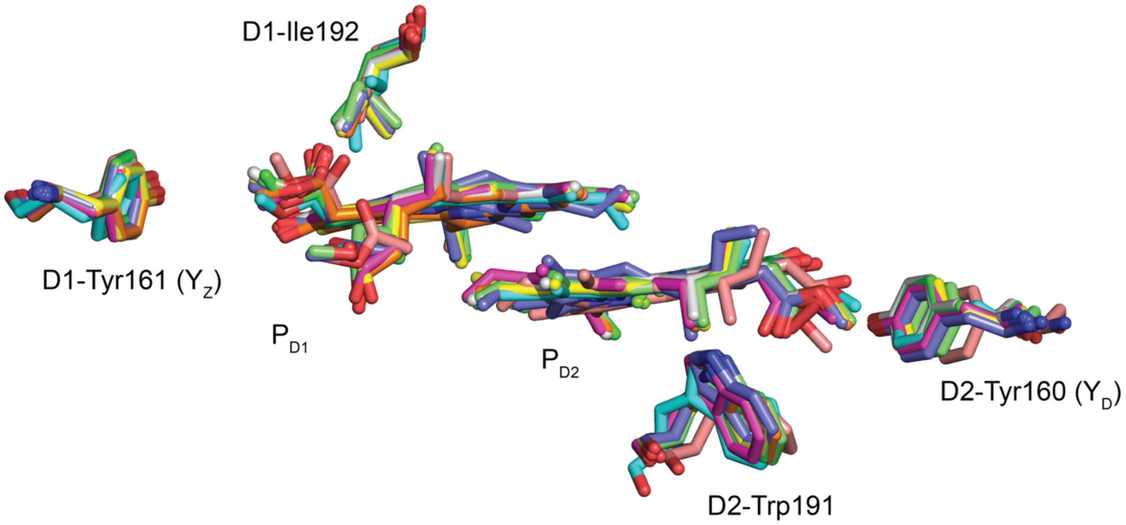

Figure S5. Positional conservation of key D1 and D2 residues across different PSII organisms (mentioned in Table S5) based on superimposed structural units. Structures are color-coded according to their PDB IDs: 3WU2, green; 1W5C, cyan; 3JCU, light magenta; 6KAC, yellow; 6YP7, salmon; 7N8O, white; 7OUI, slate; 7PI0, orange; and 8C29, lime.

References

1. Umena, Y.; Kawakami, K.; Shen, J.-R.; Kamiya, N. Crystal Structure of the Oxygen-Evolving Photosystem II at a Resolution of 1.9 Å. *Nature* **2011,** *473*, 55–60.
2. Biesiadka, J.; Loll, B.; Kern, J.; Irrgang, K.-D.; Zouni, A. Crystal structure of cyanobacterial photosystem II at 3.2 Å resolution: a closer look at the Mn-cluster. *Phys. Chem. Chem. Phys.* **2004,** *6*, 4733–4736.
3. Grinzato, A.; Albanese, P.; Marotta, R.; Swuec, P.; Saracco, G.; Bolognesi, M.; Zanotti, G.; Pagliano, C. High-Light versus Low-Light: Effects on Paired Photosystem II Supercomplex Structural Rearrangement in Pea Plants. *Int. J. Mol. Sci.* **2020,** *21*, 8643.
4. Gisriel, C. J.; Wang, J.; Liu, J.; Flesher, D. A.; Reiss, K. M.; Huang, H.-L.; Yang, K. R.; Armstrong, W. H.; Gunner, M. R.; Batista, V. S.; Debus, R. J.; Brudvig, G. W. High-resolution cryo-electron microscopy structure of photosystem II from the mesophilic cyanobacterium, Synechocystis sp. PCC 6803. *Proc. Natl. Acad. Sci. U. S. A.* **2022,** *119*, e2116765118.
5. Graça, A. T.; Hall, M.; Persson, K.; Schröder, W. P. High-resolution model of Arabidopsis Photosystem II reveals the structural consequences of digitonin-extraction. *Sci. Rep.* **2021,** *11*, 15534.
6. Opatíková, M.; Semchonok, D. A.; Kopečný, D.; Ilík, P.; Pospíšil, P.; Ilíková, I.; Roudnický, P.; Zeljković, S. Ć.; Tarkowski, P.; Kyrilis, F. L.; Hamdi, F.; Kastritis, P. L.; Kouřil, R. Cryo-EM structure of a plant photosystem II supercomplex with light-harvesting protein Lhcb8 and α-tocopherol. *Nat. Plants* **2023,** *9*, 1359–1369.
